# Whole-Body Human Ultrasound Tomography

**DOI:** 10.21203/rs.3.rs-4714949/v1

**Published:** 2024-07-17

**Authors:** David C. Garrett, Jinhua Xu, Yousuf Aborahama, Geng Ku, Konstantin Maslov, Lihong V. Wang

**Affiliations:** Caltech Optical Imaging Laboratory, Andrew and Peggy Cherng Department of Medical Engineering, Department of Electrical Engineering, California Institute of Technology, Pasadena, CA 91125 USA

## Abstract

Ultrasonography is a vital component of modern clinical care, with handheld probes routinely used for a variety of applications. However, handheld ultrasound imaging is limited by factors such as the partial-body field of view, operator dependency, contact-induced distortion, and lack of transmission contrast. Here, we demonstrate a new system enabling whole-body ultrasound tomography of humans in reflection and transmission modes. To generate 2D isotropically resolved images across the entire cross-section *in vivo,* we use a custom 512-element circular ultrasound receiver array with a rotating ultrasonic transmitter. We demonstrate this technique in regions such as the abdomen and legs in healthy volunteers. We also showcase two potential clinical extensions. First, we readily observe subcutaneous and preperitoneal abdominal adipose distributions in our images, enabling adipose thickness assessment over the body without ionizing radiation or mechanical deformation. Second, we demonstrate an approach for rapid (seven frame-per-second) biopsy needle localization with respect to internal tissue features. These capabilities make whole-body ultrasound tomography a potential practical tool for clinical needs currently unmet by other modalities.

## Introduction

Since its inception in the mid-20^th^ century, ultrasound imaging has revolutionized healthcare by providing rapid and affordable insight into tissue structure and function. Early systems employed single transducers scanned linearly or circularly with subjects immersed in a water bath [1], [2], later followed by membranes or articulating arms to image regions in the abdomen [3], [4]. Initial results were promising for disease diagnosis [5], but imaging required mechanical scanning over ~1 hour [6]. Later developments in transducers and electronics led to linear probes [7], where multiple channels could be used in parallel. The handheld probe remains the most used form of ultrasonography and has found many clinical applications. However, probes require trained operation [8], have limited ability to visualize features behind bone or air pockets, and provide only reflection-mode images over a narrow field of view (FOV). The FOV can be expanded by scanning the probe around the periphery of a region like the human thigh and co-registering adjacent frames [9], [10]. However, current approaches require manual probe movement to maintain contact with the skin, which can lead to image variation between operators and systems [11], [12].

More recently, alternate approaches using smaller immersion tanks with planar [13], linear [14], ring [15], or hemispherical [16] transducer arrays have been investigated for ultrasound tomography (UST) imaging of the breast [17] or limbs. These systems record both reflected and transmitted signals, allowing for the generation of reflectivity, speed of sound, and attenuation coefficient profiles. In extending to human-scale imaging, acoustically opaque regions like bone or air pockets have been typically viewed as insurmountable challenges. Nevertheless, a recent study achieved whole-body imaging of piglets despite the presence of bone and air [18], and another recent system enables volumetric reflection-mode imaging of vasculature and bones in human extremities like the arm [19]. However, these system geometries and parameters (e.g., acoustic frequency, transmitter power, and detection sensitivity) are not yet suitable for whole-body human imaging.

In this work, we developed a system that enables whole-body UST imaging of humans immersed in water, resulting in 2D isotropic images of reflectivity, speed of sound, and attenuation coefficient profiles. We constructed a custom 512-element receiver ring array, combined with a single-element transmitter that rotates around the subject. To image deep in the body, we enhance the signal sensitivity by using low-noise parallel preamplifiers directly coupled with the receiver array and by exciting the transmitter with a chirp waveform. Compared to handheld probes, we reduce issues of acoustic shadowing from regions containing bone or air pockets by using full 360° viewing angles. In comparison to MRI and other standard imaging modalities, whole-body UST is a potential low-cost, safe, and convenient tool for screening and monitoring abdominal conditions.

We demonstrate this technique by imaging regions in the abdomen and legs in healthy volunteers, where several organs and key features are clearly observed in reflection-mode images, and profiles of tissue speed of sound and attenuation coefficient are obtained. Furthermore, we can observe abdominal adipose layers in these images, making UST an appealing technique for assessing adipose thickness distributions. We also demonstrate an approach for localizing biopsy needles deep in tissue with respect to internal features. By coupling an acoustic transmitter to a commercial needle and detecting the resulting scattered signals from the needle tip, we obtain seven frame-per-second images of the needle tip location. When combined, these techniques showcase whole-body UST as a safe and practical modality for a variety of clinical applications.

## Results

### Whole-body imaging

**Fig. 1.**
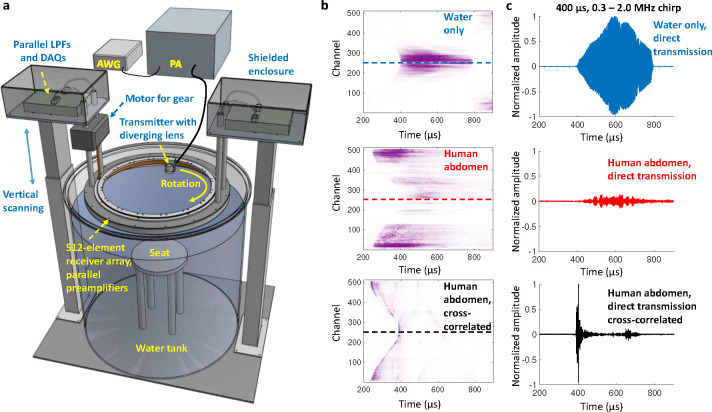
Whole-body UST system. **a** System diagram. AWG: arbitrary waveform generator; PA: power amplifier; LPFs: low-pass filters; DAQs: data acquisition modules. **b** Example signals recorded with the receiver array. Top: water only in tank. Middle: human abdomen. Bottom: human abdomen after cross-correlation with the water only signals. The dashed line indicates the channel shown in panel **c**. **c** Example signals from an individual receiver channel directly opposite the transmitter.

We developed a custom 60 cm diameter, 512-element acoustic receiver array with 1 MHz center frequency. A 1.5-inch diameter 2.25 MHz transducer (Olympus V395) with a custom cylindrical diverging polymethylpentene (TPX) lens is used as a transmitter. The transmitter is mounted on a plastic gear that rotates around the subject using a stepper motor. All receiver channels are preamplified using custom circuit boards inside the array (Supplementary Fig. 1), and these signals are digitized in parallel using two data acquisition modules (DAQs, Photosound Legion) inside shielded enclosures. The array is mounted on two vertical motor stages to adjust its height in a water immersion tank. Water acts as acoustic coupling between the skin and transducers. Anarbitrary function generator (Siglent SDG2042X) connected to a 300-Watt RF power amplifier (ENI A300) excites the transmitter. The system hardware is shown in Fig. 1a.

To enhance the signal-to-noise ratio (SNR) without exceeding the mechanical index (MI) safety standard, we use a 400 μs chirp signal spanning 0.3 – 2.0 MHz. We first record the transducer response using only water in the imaging domain, which is then cross-correlated with the target response to recover a pulse-like representation. Example signals from the receiver array for water and the human abdomen are given in Fig. 1b, showing both backscattered and transmitted signals recorded in parallel. Individual traces for the channel opposite the transmitter (channel 256) are given in Fig. 1c. This approach also enables channel calibration using the water scan, where the same receive amplitude is expected for channels directly opposite the transmitter.

**Fig. 2.**
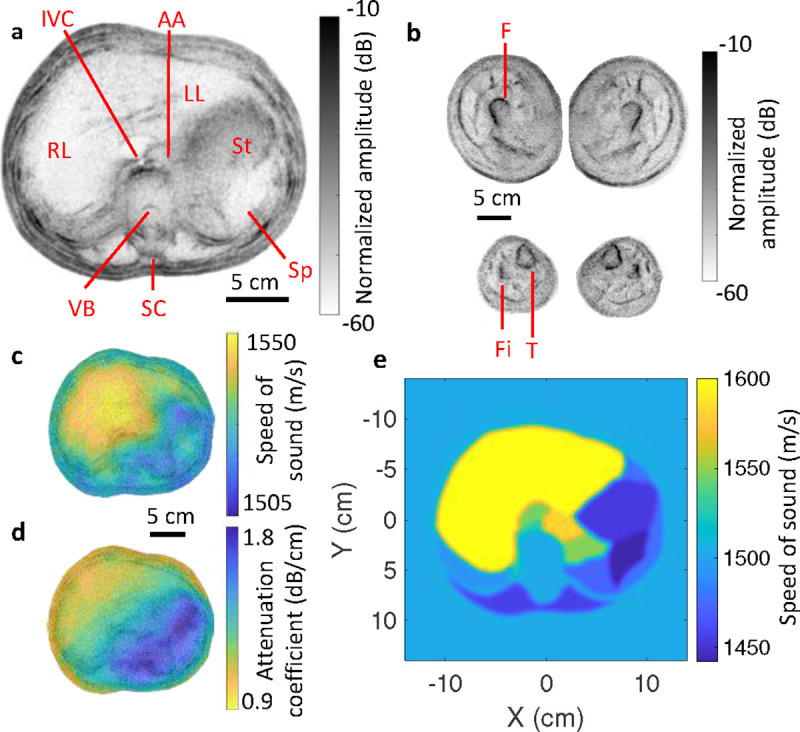
Whole-body UST of a healthy female. **a** Reflectivity image of human abdomen. IVC: inferior vena cava. AA: abdominal aorta. RL: right lobe of liver. LL: left lobe of liver. VB: vertebral body. SC: spinal cord. St: stomach. Sp: spleen. **b** Reflectivity image of human upper leg (top) and lower leg (bottom). F: femur. Fi: fibula. T: tibula. **c** and **d** show the speed of sound and attenuation coefficient profiles, respectively, overlaid on the reflectivity image. **e** Speed of sound reconstruction constrained using organ regions determined from the reflectivity image.

We demonstrate whole-body UST with healthy volunteers. For abdominal imaging, the subject sits on a stool in the water immersion tank with their head held against a cushion to reduce motion, and with their arms raised slightly to lift the ribs. During a 10-second scan, the subject is asked to remain still and to hold their breath. Fig. 2a shows an example reflection-mode image of the abdomen of a 24-year-old female. The image is displayed in inverse grayscale (brighter regions are more anechoic) normalized to the peak pixel amplitude. The outer boundary is extracted using an automated segmentation tool [20]. Various structures are visualized, including the liver, stomach, spleen, abdominal aorta, and vertebral body. Note that despite the presence of bone and air pockets, our geometry enables imaging of regions deep in the body. Due to our lower acoustic frequency than typical probe-based ultrasonography, our images correspond primarily to reflections from tissue boundaries rather than from scattering within tissues [21]. With the same subject standing in the immersion tank, we also imaged the legs as shown in Fig. 2b. In the upper legs, the femur, surrounding muscle groups, and adipose boundaries are observed. The tibia and fibula are visualized in the lower legs as well as adipose boundaries.

We also use the signals transmitted through the body to reconstruct profiles of the speed of sound and attenuation coefficient, which are overlaid on the reflection-mode images in Fig. 2c and d. We observe a higher speed of sound in the liver compared to other organs, as expected from literature values [22], and greater attenuation coefficient in the spine and stomach, which may contain air. Since we obtain distinct organ boundaries from the reflection-mode image, we can also segment organ contours to constrain the transmission-mode reconstruction for bulk organ regions as shown in Fig. 2e. We expect negligible transmission through bones like the vertebral body, so we do not solve for their speed of sound. We otherwise find good agreement with literature values for various organs such as the liver (Supplementary Fig. 8). This supports this approach as a potential tool for quantitatively evaluating conditions like liver fibrosis or non-alcoholic fatty liver disease [23].

**Fig. 3.**
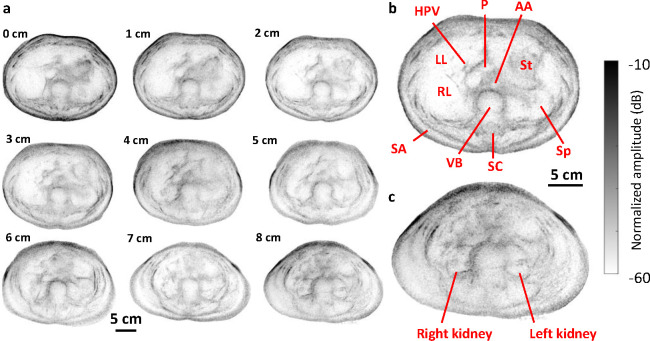
Example reflection-mode UST images of a healthy female’s abdomen. **a** Labels denote distance downward from the ribcage. **b** Expanded view of the 3 cm image. **c** Expanded view of the 8 cm image. HPV: hepatic portal vein. IVC: inferior vena cava. AA: abdominal aorta. RL: right lobe of liver. LL: left lobe of liver. St: stomach. SA: subcutaneous adipose. VB: vertebral body. SC: spinal cord. Sp: spleen.

With another female volunteer, we performed scans at 1 cm vertical intervals from approximately the ribcage to the pelvis. The subject was in the immersion tank for approximately 10 minutes over the entire imaging session. Example 2D images are shown in Fig. 3. Additional features are observed in some slices, such as the pancreas, hepatic portal vein, and kidneys. The entire liver cross-section is also visualized, making whole-body UST a potential tool for evaluating liver health.

### Adipose thickness assessment

Subcutaneous adipose (SA) and preperitoneal adipose (PA) distributions are important indicators of metabolic health [24]. SA thickness can be measured with calipers and probe-based ultrasound. Calipers are known to be less accurate for individuals with larger SA thickness, are operator-dependent, and have only moderate agreement with estimates from MRI [25]. Probe-based ultrasound has been found to be more accurate and also enables PA thickness measurement [26], but requires operator training and is affected by the pressure applied on the skin by the probe [27]. These methods also require repositioning of the instrument at each measurement location, so they are not convenient for determining whole-body adipose distributions [28].

**Fig. 4.**
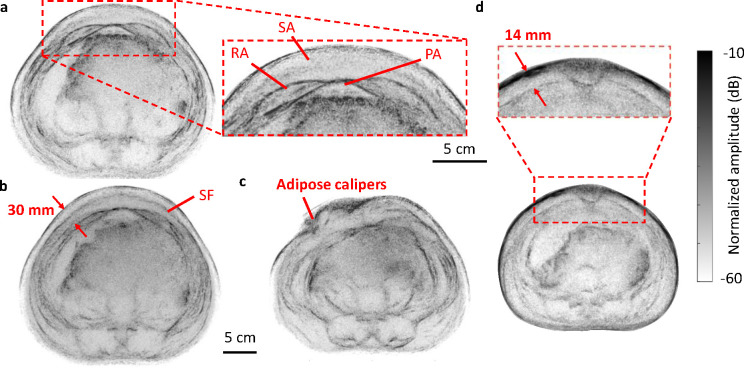
Abdominal adipose thickness assessment of healthy volunteers. **a** UST image of the entire body of a male volunteer, with an inset showing adipose regions in the anterior region of the abdomen. **b** Abdominal image showing Scarpa’s fascia in SA. **c** UST image with adipose calipers positioned on the abdomen. RA: rectus abdominus. SA: subcutaneous adipose. PA: preperitoneal adipose. SF: Scarpa’s fascia. **d** UST image of a female volunteer. SA thickness is evaluated in the image where calipers were used.

Whole-body UST is an appealing approach for SA assessment since it can clearly visualize the adipose layer around the abdomen periphery without mechanical deformation. To demonstrate this, we imaged a subject with greater SA thickness (27-year-old male). The resulting reflectivity image is shown in Fig. 4. On the anterior side of the abdomen, the SA and PA regions are observed with respect to the skin surface and rectus abdominus muscles. We additionally observe two distinct layers in the SA: the superficial and deep adipose layers, separated by the superficial (or Scarpa’s) fascia [29], [30], [31], as shown in Fig. 4b. Note that internal features are less well visualized due to the greater tissue depth and potential increased air content in organs. Adipose layers are also observed on the posterior of the body.

To assess the use of calipers for individuals with greater SA thickness, we obtained a UST image while performing a caliper measurement. As seen in Fig. 4c, the calipers do not capture the entire SA thickness [32]; our caliper measurement was 19 mm, compared with approximately 30 mm from the UST image. We also imaged a subject with thinner SA (25-year-old female) to validate UST thickness estimates. As shown in Fig. 4d, our estimate of 14 mm corresponds more closely with the caliper measurement of 13 mm used at the same location. Since whole-body UST is fast, safe, and lower cost than MRI, it may be a useful tool for guiding and assessing weight loss regimes, clinical trials for weight loss drugs, or liposuction treatment [33].

### Biopsy needle localization

We next demonstrate UST-guided biopsy needle localization. In clinical practice, when a region is suspected of being cancerous, a small sample of tissue is collected using an inserted needle. To guide the positioning of the needle with respect to internal features, ultrasound and X-ray CT imaging are often used [34]. However, probe-based ultrasound localization is generally only used for superficial targets like in the breast, and it requires that the needle is approximately orthogonal to the imaging probe to provide sufficient backscatter. To improve the ultrasound visibility, some manufacturers use treatments like scoring or bubble-filled polymer coatings to generate more isotropic scattering. However, these approaches can increase the insertional friction of the needle [35]. CT needle guidance enables whole-body localization, but it requires iterative positioning and leads to harmful radiation to the patient.

**Fig. 5.**
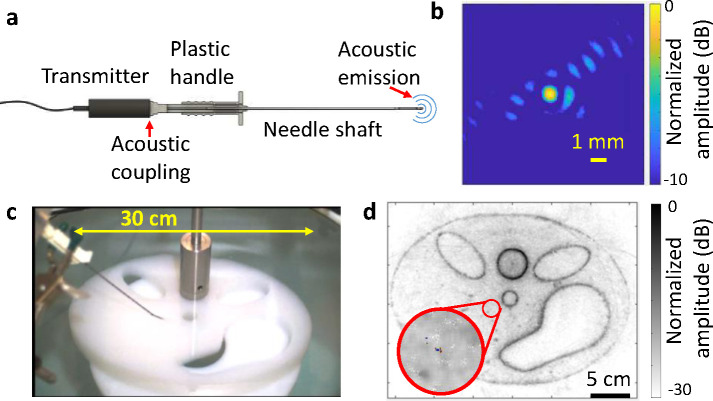
UST biopsy needle localization. **a** Diagram of needle configuration. **b** Representative image of the needle’s acoustic response in water. **c** Video frame showing the needle inserted into an agarose phantom. **d** Reconstructed video frame overlaid on a reflectivity image. The red circle is automatically placed around the center acoustic response.

In our approach, we use a commercial core biopsy needle consisting of a solid stainless-steel core (1.5 mm diameter) that translates within a hollow sleeve. We found that without modifying the needle, ultrasound signals can be coupled into the needle tip from the plastic handle. These signals propagate down the needle like an acoustic waveguide, and they are scattered isotropically at the needle tip (Fig. 5a). To improve SNR, we used the same chirp signal as in UST. The scattered signals from the tip are detected with our acoustic array and are cross correlated with the water chirp response. The propagation time along the needle is calibrated for and remains constant. We expect the dominantly excited mode to be longitudinal with approximately constant phase velocity over our frequency range (Supplementary Fig. 8).

We then determine the location of the needle tip by performing one-way backprojection after accounting for the propagation time down the length of the needle. An example image response from the needle is given in Fig. 5b. The image FWHM is found to be ~0.7 mm. Using this approach, we achieve ~7 frame per second needle localization over a human-scale FOV. An example video frame is shown in Fig. 5d, where the needle response is overlaid on a UST reflectivity image of an agarose phantom held with a steel post. The needle’s center response is automatically determined based on the maximum image amplitude (if greater than a fixed threshold). The full video is given in Supplementary Video 1. As seen, the acoustic image quickly and accurately tracks the location of the needle tip with respect to the phantom, even when moving quickly or being inserted into the phantom.

## Discussion

We developed a system for whole-body ultrasound imaging. Compared with clinical handheld-probe-based ultrasonography, our approach images cross-sections of the entire human body and visualizes three contrasts: reflectivity, speed of sound, and attenuation coefficient. Furthermore, clinical ultrasonography typically requires trained operation for observing regions of interest. Our approach could be automated since it requires minimal subject positioning. This could be an appealing feature for applications requiring frequent imaging and would help reduce cost compared to other modalities.

Whole-body UST could also be of clinical use for screening organ size or structure as an indicator of inflammation or disease [36], which would be evaluated in further clinical studies. For instance, liver cirrhosis may be visualized and tracked over time to monitor its progression. Aortic aneurysms may also be visualized with UST in critical care patients. The speed of sound and attenuation coefficient could be used as diagnostic tools, for instance, to assess changes due to non-alcoholic fatty liver disease [36]. Treatments like shockwave lithotripsy may also benefit from our speed of sound maps to improve acoustic focusing for treatment of kidney or gallbladder stones.

Compared with other emerging techniques like low-field MRI [37], whole-body UST is faster (~10 seconds per 2D slice) with comparable or finer resolution (~1 mm), and it does not require a shielded room or magnet-compatible environment. Further, it is more portable, more open, and less noisy than MRI. Also, due to its magnet-free operation, it can be used for subjects with implants that are incompatible with MRI. When combined, these features make whole-body ultrasound tomography a potential practical tool for clinical needs currently unmet by other modalities.

Adipose assessment over the entire body takes advantage of our large FOV. Compared with calipers or handheld ultrasound, this approach visualizes subcutaneous and preperitoneal adipose around the entire periphery without mechanical deformation. This could be an appealing tool for liposuction planning and evaluation, weight loss monitoring, or pharmaceutical trials for anti-obesity drugs, where MRI and CT are prohibitively costly or harmful. Improved image quality or contrast may further enable the evaluation of visceral adipose regions. Muscle regions are also observed in our images (e.g. abdominal and leg muscles), which may be useful for guiding athletic training.

Whole-body UST could be used in applications such as image-guided needle biopsy where CT imaging is conventionally used. With our whole-body FOV, the location of the biopsy needle can be localized with respect to internal features without use of ionizing radiation. Whereas CT guidance requires iterative needle positioning and imaging, UST could enable real-time feedback. This technique could also be used for localization with minimally invasive surgical robots [38]. Here, the needle or tool could be tracked with respect to internal features while performing a procedure which may deform tissues.

To be more practical in clinical applications, several improvements could be made to this system. Water immersion for acoustic coupling could be avoided by using inflatable water bags like those used in shockwave lithotripsy [39]. Our 10-second imaging time is currently limited by both the mechanical scanning rate of the transmitter and the DAQ transfer rate (repetition rate and acquisition length). A faster scanning rate could be achieved using a slip ring for electrical connection to the transmitter (like those used in CT systems) and a more powerful driving motor. The roundtrip acoustic propagation time within the immersion tank is approximately 1 ms. Therefore, the repetition rate could be increased to 1 kHz from our current device limit of 180 Hz. An acoustic array could also be used to transmit and receive signals, but this may reduce sensitivity due to electrical switches and limited chirp signal length and quality.

In the future, we also plan to enhance this system with additional photoacoustic and thermoacoustic contrast. Using the same acoustic receivers, these images could be immediately co-registered with our UST images to overlay optical and microwave absorption profiles. We also aim to improve our transmission-mode reconstruction quality using techniques such as full-wave inversion [40] to better localize variations in the speed of sound and attenuation coefficient. Additional acoustic elements could also reduce image acquisition time and provide 3D imaging capability.

## Materials and Methods

### System hardware

We developed a custom 512-element, 60 cm diameter acoustic receiver array with 1 MHz center frequency. This geometry is scaled from similar systems for small animal or human breast photoacoustic imaging [41], [42]. We use lower acoustic frequencies than typical handheld probes or breast UST systems to enable whole-body imaging. For instance, typical acoustic attenuation of ~1 dB⋅cm^−1^⋅MHz^−1^ results in ~30 dB attenuation across a typical 30 cm diameter human cross-section at 1 MHz [21].

All 512 receiver array elements are 1 mm thick, 3 mm × 10 mm gold-coated piezoelectric polymer (PVDF-TrFE, PolyK Technologies LLC). We chose PVDF-TrFE for its broad bandwidth and ease of manufacturing since it is more closely acoustically matched to water than other piezoelectric materials. Each element is capacitively coupled to copper cladded polyimide electrodes by bonding with high-strength epoxy. A continuous copper cladded polyimide electrode is used for the ground reference. The electrodes are then directly connected to parallel preamplifiers implemented on custom annular printed circuit boards. The preamplifiers provide 15 dB voltage gain with 100 kΩ input impedance.

**Fig. 6.**
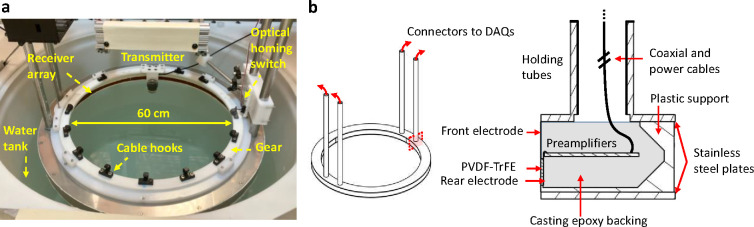
Whole-body UST hardware. **a** System photograph. **b** Acoustic receiver array design, showing a cross-section of the array. DAQs: data acquisition modules.

A 60 cm diameter plastic disc was machined and used as a mold for the inner surface of the array. The elements and preamplifiers are housed in a stainless-steel shielded enclosure, with coaxial cables for each element connected through stainless steel holding tubes. Casting epoxy is used as a backing material for each element, and an angled back panel is used to reduce reverberation (Fig. 6). All channels are low-pass filtered (*f*_*c*_ = 2 MHz) and digitized (Photosound Legion) in parallel at 5 MSPS. The digitizers are controlled and transfer data through USB over optical fiber to reduce interference. The preamplifiers are powered by rechargeable lithium polymer batteries with a DC voltage regulator to reduce electrical noise. To account for geometrical error during manufacturing, the technique described in [43] is used to calibrate each element’s position.

The gear rotation is driven with a stepper motor. An optical homing switch is used to ensure a consistent initial rotation angle. Plastic hooks are mounted on the gear to hold the transmitter cable on the gear surface during rotation (Supplemental Video 2).

### Acquisition parameters

To enhance the signal-to-noise ratio (SNR) while limited by the mechanical index, a linear chirp signal versus time (t) is used with a time varying frequency $f(t)=f_{r}t+f_{0}$, where $f_{r}=\left(f_{1}-\right.\left.f_{0}\right)/T$ is the linear chirp rate, $f_{0}=0.3\;MHz$ is the lower frequency, $f_{1}=2.0\;MHz$ MHz is the upper frequency, and T=400μs is the chirp duration. The transmitted frequencies are limited by the bandwidths of the transmitter and receivers. We used a maximal pulse duration given our maximal acquisition time of 800 μs, allowing for recovery of the roundtrip reflected signals over the entire FOV. The resulting transmitted chirp signal is

(1) $$x\left(t\right)=\sin\left[2\pi \left(\frac{f_{r}}{2}t^{2}+f_{0}t\right)\right].$$

Compared to a pulse with similar peak pressure, this results in an expected SNR gain of $\sim \sqrt{T\cdot B}$, where $B=f_{1}-f_{0}$ is the acoustic bandwidth [44]. In addition to the target, we also perform a scan with only water in the imaging domain, resulting in recorded signals $x_{w,i}(t)$ for each receiver element i. This provides the response of each transducer to the chirp which is then cross-correlated with the target’s chirp response $x_{c,i}(t)$. The pulse response for the target signals $\chi_{s,i}(t)$ is then recovered for each element i as:

(2) $$\chi_{s,i}\left(t\right)=\frac{x_{w,i}\left(t\right)\;⋆\;x_{c,i}\left(t\right)}{max\left[x_{w,i}\left(t\right)\;⋆\;x_{w,i}\left(t\right)\right]},$$

where ⋆ denotes cross-correlation. We normalize by the maximum of the autocorrelation of $x_{w,i}(t)$ to account for sensitivity variation in the receiver elements. The transmitter operates with a pulse repetition rate of 180 Hz. With the gear rotation time of 10 seconds, this results in 1800 transmitted pulses over a full circular scan around the target.

### Image quality

**Fig. 7.**
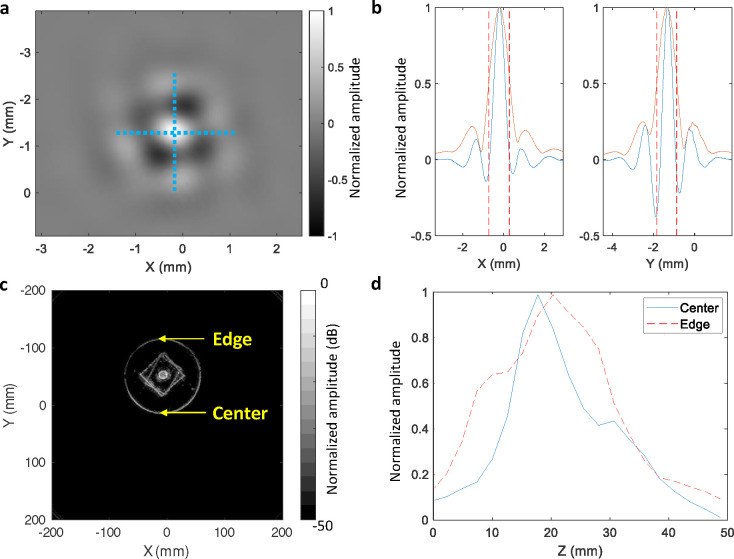
In-plane and elevational resolution assessment. **a** Reconstructed reflectivity image of a thin wire. **b** Profiles along the x and y axes: pixel amplitude (blue), the magnitude of its Hilbert transform (orange), and the FWHM (dashed vertical lines). **c** Example reflectivity image of a brass disc used for elevational resolution assessment. **d** Profiles of the center and edge responses at different z positions.

We assessed our in-plane resolution using a thin (< 0.1 mm) metallic wire. The reconstructed image and axis profiles are given in Fig. 7a and b. We find an in-plane FWHM of approximately 0.9 mm. To determine our elevational resolution, we imaged a thin brass disc positioned such that its edges were at the center and outer boundary of our typical FOV (Fig. 7c). The height of the disc was scanned, and a UST image was obtained at 2.5 mm increments. An elevational FWHM of 15 mm and 25 mm was found for the center and edge of the FOV, respectively. Neither the transmitter nor receivers are focused in the elevational direction, but their larger dimensions in this direction reduces their acceptance angle.

### Human imaging protocol

Three volunteers (two female, one male) consented to being imaged in this system. This imaging procedure was approved by the Caltech Institutional Review Board (protocol IR21–1099). Prior to human imaging, we used a calibrated hydrophone (Onda HGL-0085) positioned immediately in front of the transmitter to evaluate the mechanical index as less than 0.2, whereas the limit from the U.S. Food and Drug Administration is 1.9 [45]. After the patient entered the water tank, we asked them to use their hand to wipe away any air bubbles that may have accumulated on their abdomens.

## Data Availability

The data that support the findings of this study are provided within the paper and its Supplementary materials. The reconstruction algorithm and data processing methods can be found in the paper. The reconstruction code is not publicly available because it is proprietary and may be used in licensed technologies.
